# The use of virtual reality and augmented reality in psychosocial rehabilitation for adults with neurodevelopmental disorders: A systematic review

**DOI:** 10.3389/fpsyt.2022.1055204

**Published:** 2022-12-14

**Authors:** Bhing-Leet Tan, Jing Shi, Suyi Yang, Hannah Loh, Desiree Ng, Cherie Choo, Alice Medalia

**Affiliations:** ^1^Health and Social Sciences Cluster, Singapore Institute of Technology, Singapore, Singapore; ^2^Occupational Therapy Department, Institute of Mental Health, Singapore, Singapore; ^3^Department of Psychiatry, New York State Psychiatric Institute, Columbia University Vagelos College of Physicians and Surgeons, New York, NY, United States

**Keywords:** psychosocial rehabilitation, neurodevelopmental disorders, functional outcomes, community living, employment, social participation, systematic review, virtual reality and augmented reality

## Abstract

**Objectives:**

Virtual reality and augmented reality have been used in psychosocial rehabilitation for adults with neurodevelopmental disorders in recent years, to provide functional training in a scaffolded and appealing manner. This systematic review attempted to evaluate (1) how virtual reality or augmented reality technology was deployed, when used as an intervention for adults with neurodevelopmental disorders; and (2) how virtual or augmented reality-assisted psychosocial rehabilitation programs impacted on the functional domains of community living, employment and social participation.

**Methods:**

The Preferred Reporting Items for Systematic Reviews and Meta-Analyses (PRISMA) guidelines was adopted and a search of publications between June 2012 and June 2022 was carried out. The target groups were adults with schizophrenia/schizoaffective disorders, autism spectrum disorder, intellectual disabilities and attention deficit hyperactivity disorder. Interventions that targeted at least one functional domain were included.

**Results:**

The database search generated 1,267 records and 38 studies met the inclusion criteria. Three studies utilized augmented reality while the rest utilized virtual reality. The virtual scenarios were displayed in various ways, such as head-mounted displays, computer screens, mobile devices and cave rooms. A few studies also used features such as speech recognition, eye tracking and motion-capture device to provide real-time feedback to participants during rehabilitation. Eleven studies reported interventions that addressed community living, 15 studies addressed vocational skills and nine studies trained participants in social skills or social cognition. Three studies sought to improve quality of life using virtual scenarios to expose participants to various situations. Majority of these studies reported preliminary promising results, with improvement in the functional domains addressed. However, several studies had small sample sizes and many single-arm pretest-posttest studies were assessed to be of serious or critical risk of bias.

**Conclusion:**

Virtual reality and augmented reality are deployed in various ways to augment psychosocial rehabilitation for adults with neurodevelopmental disorders. Most interventions target skills training or strategy learning in the areas of community living, work and social participation. Preliminary positive findings of their effects on functional performance were reported. Larger and robust studies using ecologically valid outcome measures will be needed to establish their effects on real-world functional outcomes.

**Systematic review registration:**

identifier: CRD42022335443.

## Introduction

Psychosocial rehabilitation is a process of facilitating persons with psychiatric disabilities to reach their optimal level of functioning in their natural settings, as well as to enhance their sense of empowerment and quality of life (1). Adults with neurodevelopmental disorders such as intellectual disabilities (ID), autism spectrum disorder (ASD), and schizophrenia can benefit from psychosocial rehabilitation to maximize their participation in community living, work and leisure (2, 3). This is often done through skills training, task adaptations, environmental modifications, advocacy and educating stakeholders (2, 3).

Technology and assistive devices have often been used in psychosocial rehabilitation, due to their ability to provide structured graded training to facilitate scaffolding, as well as their interesting graphics to motivate service users (4, 5). In the recent decade, augmented and virtual reality have enabled rehabilitation practitioners to provide a more immersive training environment. Virtual reality (VR) enables stimulation of the senses by transporting the users to a realistic three-dimensional environment, while allowing users to interact with objects in the simulated environment (6). Augmented reality (AR) differs from VR in that the virtual three-dimensional graphics and images are superimposed on the real environment, such that the users are still able to perceive their surroundings (7). Both VR and AR present as viable options for rehabilitation practitioners to conduct skills training in a less time-consuming and costly way, as there is no need to set up a real training environment and the virtual consumables can be reused. In a pilot study on an AR-enabled vocational training program for persons with intellectual and developmental disabilities (IDD), participants also reported that they could experiment with new behaviors within the virtual environment in a self-paced and less anxiety provoking manner (8).

In view of these benefits, practitioners have harnessed the capabilities of VR and AR as adjunctive interventions for children and youths with ID, ASD, and attention-deficit hyperactivity disorders (ADHD). Many of these interventions are conducted in special education settings and positive findings have been found (6, 9). However, the use of VR and AR in psychosocial rehabilitation for the adult population with IDD is less established. As for adults with schizophrenia, reviews have been conducted on the use of VR and AR in the treatment of psychiatric symptoms such as hallucinations and delusions (10–12). In recent years, however, there is also an emerging interest in the use of VR and AR in psychosocial rehabilitation for schizophrenia, to improve outcomes in personal, functional and clinical recovery. It would be useful to review the effectiveness of such psychosocial rehabilitation programs in facilitating service users to achieve valued goals in life.

Therefore, this systematic review attempted to evaluate a variety of psychiatric rehabilitation programs that utilized augmented or virtual reality for adults with neurodevelopmental disorders, to understand how they address the functional domains of community living, employment and social participation. The review focused on two questions:

1. How was the virtual reality or augmented reality technology deployed, when used as an intervention for adults with neurodevelopmental disorders?
2. How did virtual reality and augmented reality-assisted psychosocial rehabilitation programs impact on the functional domains of community living, employment, and social participation?

Treatment approaches, mode of delivery and outcome measurements used in these studies were examined. In addition, their effects on addressing proximal outcomes such as cognitive performance, social skills, vocational skills or independent living skills were discussed.

## Methods

### Study design

This systematic review was registered with PROSPERO (ID: CRD42022335443) and was conducted using the Preferred Reporting Items for Systematic Reviews and Meta-Analysis (PRISMA) guidelines (13). Findings of this review were presented in a narrative approach.

### Eligibility criteria and search strategy

The target diagnostic groups of this systematic review were neurodevelopmental disorders, which included schizophrenia/ schizoaffective disorders, autism spectrum disorder (ASD), intellectual disabilities (ID) and attention deficit hyperactivity disorder (ADHD). In some studies, intellectual disabilities and autism spectrum disorders were grouped together and known as intellectual and developmental disabilities (IDD). At least half of the study participants had to be diagnosed with these conditions and co-morbid psychiatric and/or medical conditions were included. In addition, at least half of the research participants were above 18 years of age. Other eligibility criteria were showed below.

Inclusion criteria:

- Publications in English language.
- Publications from June 2012 to June 2022.
- Types of publications: randomized controlled trials, non-randomized experimental studies, quasi-experimental studies, pretest–posttest intervention, mixed methods studies, pilot trials, and feasibility studies.
- Interventions included the use of virtual reality or augmented reality.
- Interventions addressed at least one functional outcome (self-care/daily living, community living, work/education, leisure, and social participation).

Exclusion criteria:

- Non-English publications.
- Types of publications: case reports, single case designs, study protocols, reviews, and conference papers.
- More than half of the research participants were below 18 years of age.
- Fewer than half of the research participants had a diagnosis of schizophrenia/schizoaffective disorders, autism spectrum disorders, intellectual disabilities or attention deficit hyperactivity disorders. Studies solely on formal or informal caregivers of these persons were also excluded.
- Virtual reality or augmented reality not used.
- Interventions did not target functional outcomes.

Articles included in the systematic review were identified through a computer-based search of the following databases: CINAHL, PubMed, PsychInfo, IEEE Xplore, ACM Digital Library, and Web of Science. Subject headings were used when available. Expanders and equivalent subjects were also applied. The first two authors conducted the database searches.

The search terms and Boolean operators used were: [(“virtual reality” OR “augmented reality” OR “VR” OR “AR”) AND (“psychiatric rehabilitation” OR “psychosocial rehabilitation” OR “intervention” OR “train^*^” OR “rehabilitation” OR “therap^*^” OR “functional re-training”) AND (“neurodevelopment^*^” OR “psychiatric disabilit^*^” OR “intellectual disabilit^*^” OR “developmental disabilit^*^” OR “Autism” OR “Autism spectrum^*^” OR “Asperger^*^” OR “mental retardation” OR “schizophrenia” OR “schizoaffective” OR “attention deficit hyperactivity disorder^*^” OR “ADHD”) NOT (“motor disorder^*^” OR “tic disorder^*^” OR “Tourett^*^” OR “learning disab^*^”)].

We selected literature from the past 10 years, due to the rapid technological advancements of VR and AR in the past decade, which led to changes in the way they were used by clinicians in psychosocial rehabilitation.

### Article selection and risk of bias assessments

Five authors (B-LT, CC, HL, DN, and SY) were paired up to independently assess the articles for inclusion or exclusion (B-LT was paired twice), then came together to arrive at a consensus. They also manually removed duplicates in the database searches. In addition, the reference sections of reviews and articles identified from database searches were studied by the first author for relevant citations. All authors then completed the systematic review table of the selected studies and cross-checked each other's risk of bias assessments.

#### Risk of bias assessments

The risk of bias assessments were performed by the first six authors and cross-checked with each other in pairs. The Cochrane Collaboration's ROB-2 (Risk of Bias version 2) tool was used to assess the risk of bias for randomized studies (14). The effect of assignment to intervention (the “intention-to-treat” effect) was assessed. Biases were categorized into five domains, namely (1) bias due to randomization; (2) bias due to deviations from intended intervention; (3) bias due to missing outcome data; (4) bias due to measurement of outcome; and (5) bias due to selection of reported results. An overall risk of bias indicator (“low risk,” “some concerns,” or “high risk”) was also derived. The authors followed the ROB-2 guidelines to answer signaling questions and used the algorithm to estimate the level of risk for each domain and the overall risk.

The ROBINS-I (Risk of Bias in Non-randomized Studies-of Interventions) was used to assess risk of bias for all the other non-randomized studies (15). Functional performance was used as the main outcome measurement. Biases were categorized into seven domains, namely (1) bias due to confounding; (2) bias in selection of participants; (3) bias in classification of interventions; (4) bias due to deviations from intended interventions; (5) bias due to missing data; (6) bias in measurement of outcomes; and (7) bias in selection of the reported result. Similarly, the authors answered signaling questions and used the ROBINS-I evaluation table to estimate the risk in each domain and the overall risk. The overall risk was reflected as “low,” “moderate,” “serious,” “critical,” or “no information.”

## Results

### Results of screening and selection of studies

**Figure 1:**
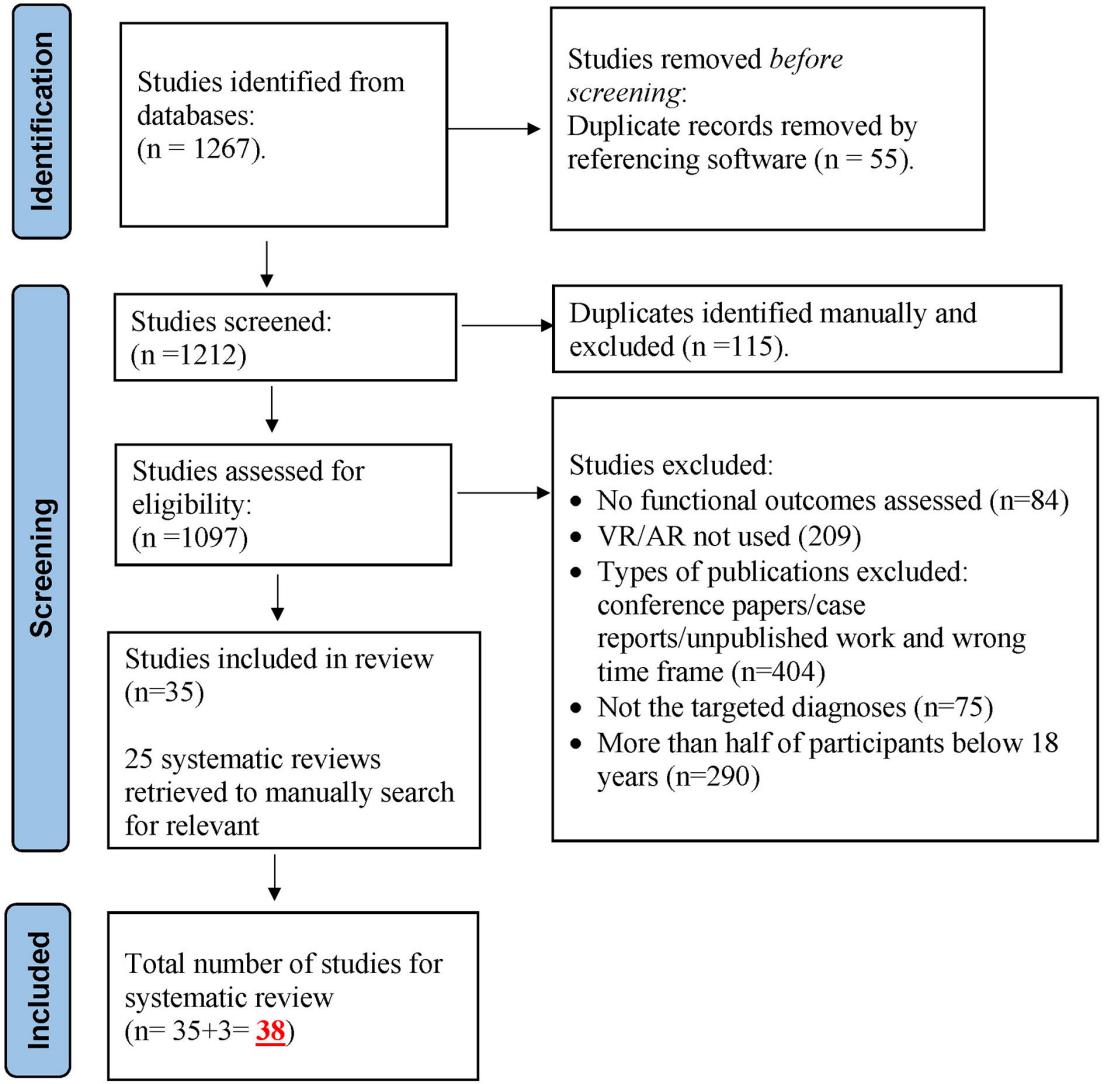
PRISMA flow diagram.

The database search generated a total of 1,267 records, out of which 55 duplicated records were identified by EndNote referencing software and excluded. The remaining 1,212 records were screened manually, and 115 duplicates were further excluded. The rest (1,097 studies) were then assessed for eligibility and a total of 1,062 studies were excluded for not fulfilling the inclusion criteria, yielding 35 selected studies for review. In addition, three studies were identified by the first author through searching the reference sections of articles manually. The final number of studies selected for this systematic review was 38. See Figure 1 for the PRISMA flow diagram.

The selected 38 studies were presented in Tables 1–4, which corresponded to the functional domains that the studies addressed, namely (1) self-care and community living; (2) vocational skills and employment; (3) social skills and social participation; and (4) quality of life. The tables outlined the study design, overall risk of bias, intervention methods, treatment modalities, target population, experimental and control conditions (if any), functional domains targeted, outcome measurements and effects of the intervention. The ROB-2 and ROBINS-I assessments were presented in the Supplementary material. An adapted robvis visualization tool format was used, with traffic light plots of estimated risk for each domain (16).

**Table 1:** Studies that addressed self-care and community living.

**Reference and country**	**Assessment of bias (ROB 2/ROBINS-I)**	**Treatment modalities/methods and therapist/trainer facilitation**	**Experimental condition^a^**	**Control condition (if applicable)^b^**	**Duration and frequency of intervention**	**Skills and/or functional domains targeted**	**Outcome measurements**	**Results/effects of intervention**
Amado et al. (17) France	ROBINS I: critical risk of bias	VR simulated town, deployed on a computer. Patients navigated as pedestrians and planned their daily activities in a virtual 3D town, using a joystick. A psychologist and an occupational therapist conducted the group sessions.	Single arm pretest-posttest design. Outpatients meeting the DSM-5 criteria for schizophrenia or schizoaffective disorders. *n* = 7 M = 38.6 (12.1) One female and seven males.	NIL	12 interactive 90-min weekly sessions.	Community living skills	Social autonomy: Social Autonomy Scale (French version). Quality of life: Schizophrenia questionnaire for Quality of life. Other scales: Self-Esteem Rating Scale, Birchwood Insight Questionnaire, WAIS-III, Battery for Assessment of Dysexecutive Syndrome.	Significant improvements in Social Autonomy Scale total scores. Improvements in attention, working memory, prospective and retrospective memory benefits, but not executive functions. Qualitative reports: better time management, planning and management of housework.
Baker-Ericzén et al. (18) United States	ROBINS I: serious risk of bias	Cognitive Behavioral Intervention for Driving (CBID): which consisted of cognitive behavioral strategies to enhance executive function, as well as driving simulation. Driving simulator consisted of a steering wheel, wide screen monitor, and gas and brake foot pedals using the STISIM Drive software. Three providers/staff and two interns trained in providing driving simulator coaching sessions.	Single arm pretest-posttest design. Teens and adults with a medical diagnosis of ASD. *n* = 19 M = 20.52 (4.40). 5% female; 95% male.	NIL	Two cohorts completed: 10 sessions (eight on content, two for assessment/feedback) Once per week for 1.5 of hours per session	Driving	Driving: Driving Cognitions Questionnaire, Driving Attitude Scale, driving simulator performance, attainment of driver's permit. Other assessments: program fidelity checklists, participant and parent satisfaction survey, semi-structured qualitative interview, State-Trait Anxiety Inventory.	Within 2 months of program, eight participants obtained a driver's permit, and one obtained a driver's license (47% demonstrated a positive functional outcome). Participants also demonstrated improvements on driving behaviors by a reduction in errors on driving violations. Statistically significant improvements were in pedestrian collisions, speeding, and centerline crossing.
Bridges et al. (19) United States	ROBINS I: serious risk of bias	AR for daily living training. AR application HP Reveal installed on iPad, with daily living task videos. During intervention, participants pointed the iPad cameras at laminated targets to activate video models, which appeared as an overlay across the target. Facilitator: research team members.	Single-arm multiple baselines across participants and behaviors design. Study conducted in dormitory at a university campus. AR app on the iPad was used to overlay the video (of making the bed, setting an alarm, and ironing) across participants' rooms. Adults with moderate to severe intellectual and developmental disabilities *n* =3 Ages 19, 20, and 36. One female and two males.	NIL	Number of sessions and duration was not provided. 9 weeks in total.	Independent Living (ironing, bed making, and setting an alarm clock).	Independent living: the number of steps the participant performed independently after viewing the HP Reveal video model. Other measurements: participant's ability to use the HP Reveal app and targets accurately 6-item questionnaire was developed to examine social validity.	Percentage of non-overlapping data and omnibus improvement rate difference for all participants was 100%. Hence, their independent living skills immediately improved.
Câmara et al. (20) Portugal	ROB2: some concerns on risk of bias	Reh@City v2.0: a virtual city where participants engaged in cognitive training (CT) through performing activities of daily living (ADLs) such as baking cookies at home and buying groceries in the supermarket. Implemented using Unity 3D game engine and installed on a desktop. Participants interacted with the virtual environment through a joystick. Conducted by psychologists.	Single-blind randomized controlled trial Reh@City v2.0 CT group. Inpatients attending psychosocial rehabilitation program. Majority had schizophrenia. *n* = 15 M = 54.07 (11.40)	Paper and pencil Task Generator (TG) group. Consists of 11 different CT tasks, such as cancellation, image pairs, problem solving, categorization etc. Inpatients attending psychosocial rehabilitation program. Majority had schizophrenia. *n* = 14 M = 49.64 (11.99)	Two 30 min sessions per week for 3 months (total 24 sessions), for both Reh@City v2.0 and Task Generator groups.	Community living skills	Quality of life: WHOQOL-BREF Cognitive assessments: MoCA, Digit Symbol, Symbol Search and Verbal Paired Associates I subtests of WAIS III, 10-min cancellation test, Rey-Osterrieth Complex Figure Test. Other assessment: BDI-II	Reh@City v2.0 group: no improvement in WHO-QOL BREF; statistically significant improvements in MoCA's total score and immediate recall. Significant reduction in BDI-II score. TG group: significant improvements in the social relationships and in the environmental domains of the WHOQOL-BREF, significantly higher scores in symbol coding, attention and word recognition.
Cox et al. (21) United States	ROBINS I: serious risk of bias	Virtual Reality Driving Simulation Training (VRDST): virtual driver's cockpit with side and rear-view mirrors and simulation presented on large, curved screen. Focus of training alternated between executive functioning driving deficits and tactical driving skills. Standard VRDST: trainer demonstrated task to the participant and then monitored participant's performance while providing continual positive verbal feedback. Automated VRDST: trainer's input was replaced by simulator's computerized voice that provided real-time auditory feedback. Eye-tracking VRDST: incorporating eye-tracking device in Standard VRDST. Videos of the eye-view during driving were shown to participants. After viewing the model video, the participant wore the eye-tracker “glasses” during driving, from which a video of his/her eye gaze during driving was produced for trainer's review.	Exploratory quasi-experimental design. Participants randomized to Standard VRDST. Eye tracking VRDST was also added to Standard VRDST. Novice drivers with ASD *n* = 14 M = 17.93 85.7% males.	Novice drivers with ASD. Participants randomized to Automated VRDST. *n* = 13 M = 17.86 85.7% males In the 2nd year, 23 participants were recruited into Routine Training. Routine Training involved giving the participants' families the state-specific Department of Motor Vehicles (DMV) training manual. Families were instructed to follow the training manual and track supervised on-road driving experience. 18 of these 23 participants were subsequently crossed over to Eye-Tracking VRDST. **Routine Training** *n* = 19 M = 17.97 73.9% males. **Eye tracking VRDST** *n* = 17 M = 18.05 72.7% males	8–12 sessions, 1-h sessions. Over 3 months.	Driving skills	In-game performance on the VRDS on tactical course and executive functioning.	The VR driving simulation significantly improved driving and executive function of novice driver with ASD (*p* < 0.01). There were no significant differences across the three VRDST groups. Only the Standard and Automated groups performed significantly better than Routine Training on the tactical simulator tests.
de la Torre-Luque et al. (22) Spain	ROBINS I: serious risk of bias	Semi-immersive virtual reality (VR) navigation training: conducted in a semi-dark room, with graphics projected from computer onto a large screen. Participants interacted using joystick and a mouse. Virtual environment displayed the ground floor of AMAPPACE center for disability, with navigation from entrance to five open rooms. Facilitated by research team.	Quasi-experimental intra-group study. *n* = 20 (13 males, seven females) M = 34.35 years (SD = 10.21). 75% of participants had intellectual disabilities. Average Andalusia Government's Disability Index of 84.95% (SD = 12.04). Participants divided into two groups: residential and day care. *N* not known for each group.	NIL	15 sessions daily, 20 min each.	Community mobility: navigation	Location tasks in the VR and in real-life building. World Health Organization- Disability Assessment Schedule II, Porteus Maze Test and Maze Stair Test.	VR location task: significant reduction in errors and time taken post-intervention. Real-life location task: significant reduction in errors and time taken post-intervention, with generalization.
Jeon et al. (23) South Korea	ROBINS I: serious risk of bias	Brush Monster: a smart toothbrush providing training based on AR that allowed the user to learn proper tooth-brushing performance to form correct tooth-brushing habits. 3D motion sensor in this brush enabled recognition of the participants' motion and provided visual guide for posture and direction. Training conducted by occupational therapist in research team.	Quasi-experimental study with non-equivalent group. Tooth-Brushing Training Based on the AR Using smart sonic toothbrush. *n* = 15, diagnosed with ID, staying in residential care facilities.	Tooth-Brushing Training using manual toothbrush and visual material, with education and practice guided by an occupational therapist. *n* = 15, diagnosed with ID, staying in residential care facilities.	Training was conducted twice a week for 12 weeks, and each session was performed for 30 min by an occupational therapist.	Self-care skills: tooth brushing, oral hygiene.	MBI (Korean version); tooth brushing time; Simplified Oral Hygiene Index.	MBI, tooth brushing time and Simplified Oral Hygiene Index: experimental group showing better improvement with statistical significance.
Miller et al. (24) United States	ROBINS I: critical risk of bias	Virtual reality air travel training (VR-ATT): virtual simulation of the steps that travelers go through in an airport. Participants were guided through entering, checking in, navigating security, waiting at the departure gate, and boarding. iPhone X and Google Cardboard (goggles) VR viewer were used to display the virtual airport. Conducted by a speech-language therapist.	Single arm pre-post pilot study. Adolescents/adults with ASD. *n* = 7 M = 18.28	NIL	VR-ATT simulation on an iPhone X and Google Cardboard once a week (20-min sessions) over 3 weeks.	Community living skills: air traveling	No standardized measurements. Participants were asked to recount the sequence of events of the simulation. They were also observed on their behavior, communication skills and any presentation of side effects.	Analyses of reports found improvements in functional language skills such as labeling vocabulary, which helped participants interact and navigate busy environments such as an airport. Participants could accurately retell the sequence of events in the virtual simulation.
Panerai et al. (25) Italy	ROBINS I: Serious risk of bias.	VR app installed on tablets to train functional skills at home (taking medicines, preparing a suitcase, shopping at the supermarket). Self-administered at home with caregivers, with chat tool to contact staff member of day services if assistance required.	Single group, pre- and post-test study Community dwelling adolescents and adults with ID receiving day services. *n* = 16 (10 females, six males) M = 26.01 (2.21)	NIL	VR training carried out at home for 11 consecutive daily sessions.	Daily living and community living skills.	*In-vivo* tests before and at the end of VR training. In-game performance virtual sessions data: number of correct task steps, number of errors, number of non-responses. Satisfaction Questionnaires (developed by research team)	*In-vivo* tests: number of correct responses increased significantly in all scenarios, while number of errors, number of prompts and time taken to execute tasks decreased significantly, apart from the suitcase task. Participant satisfaction: high level of satisfaction and minimal difficulties reported. Family satisfaction: medium to high satisfaction level.
Saiano et al. (26) Italy	ROBINS I: serious risk of bias	A virtual reality (VR) environment to train navigation and street-crossing. VR city environment was projected on a large screen and markerless motion capture device (Microsoft Kinect) was placed below the screen to record participants' full-body movements in 3D space and mapped onto virtual environment. Conducted by a therapist.	Pretest-posttest single arm study Outpatients diagnosed with autism spectrum disorder. *n* = 7 M = 29 (10)	NIL	10 sessions (1 session per week), consisting of familiarization and training phase. Familiarization (30 min per session): to practice gestures necessary to interact with the virtual environment. Training (45 min per session): to complete different paths.	Community living: navigation	Street crossing performance (by NeuroVR application): number and types of errors. Participants questionnaire: to assess their knowledge of street-crossing rules. Parents/caregivers questionnaire: to assess the participants' community navigation in everyday life.	No significant reduction in street-crossing errors, but navigation became more efficient. Participants' questionnaire: no significant reduction in number of wrong answers. Both parents and caregivers reported a significant post-treatment improvement in the subjects' performance.
Simões et al. (27) Portugal	ROBINS I: serious risk of bias	VR gamified travel training on taking public bus transportation. Participants used VR headset (Oculus Rift) and wore a bracelet for wireless electrodermal activity recording. All tasks were run on a laptop computer. Conducted by research staff at the autism center or lab.	Quasi-experimental design. Three intervention sessions, with different difficulty levels and experiences of traveling from initial location to final destination. Adults with ASD. Three participants did not use headset due to visual problems. *n* = 10 M = 18.8 (4.5)	Control group received one session of the intervention Typical adults matched by age. One participant did not use headset due to visual problem. *n* = 10 M = 21.9 (3.6)	Three sessions with duration of 20–40 min each.	Community mobility: taking the bus.	In-game measurements were used. Actions Accuracy: number of correct actions of each step of the task. Debriefing Accuracy: participants to describe the step-by-step process of riding a bus and accuracy was calculated. Task Duration: time taken to complete the task in each session. Anxiety level: measured by electrodermal activity.	Statistically significant increase in Debriefing Accuracy and significant reduction in anxiety. Increase in Action Accuracy was not significant. Statistically significant difference between the two groups in Actions Accuracy, Debriefing Accuracy and Task Duration, showing that the game could differentiate performance between participants with ASD and typical population.

**Table 2:** Studies that addressed vocational skills and employment.

**Reference and country**	**Assessment of bias (ROB 2/ROBINS-I)**	**Treatment modalities/methods and therapist/trainer facilitation**	**Experimental condition^a^**	**Control condition (if applicable)^b^**	**Duration and frequency of intervention**	**Skills and/or functional domains targeted**	**Outcome measurements**	**Results/effects of intervention**
Bozgeyikli et al. (28) United States	ROBINS I: critical risk of bias	Virtual Reality system For Vocational Rehabilitation of individuals with disabilities (VR4VR): closed-loop adaptive VR-based vocational training platform, to train six transferrable vocational skills. Three of the six skill modules (cleaning, shelving, and environmental awareness) were designed to be performed with the HMD while the remaining three skill modules (loading the back of a truck, money management, and social skills) were designed to be performed on a 180° curved screen. Facilitated by professional job trainers with a remote-control interface on a separate tablet.	Quasi-experimental small sample study. Both groups went through the six modules in VR4VR. High functioning individuals with ASD who were receiving vocational rehabilitation services. *n* = 9 M = 29 (9.02). Sex not stated.	Control sample were neurotypical individuals without ASD. They went through the same six modules in VR4VR, except they completed the modules in a single session of 4 h. *n* = 9 M = 25.44 (6.26). Sex not stated.	Two sessions. 2 h per session.	Vocational skills	Vocational skills: 1-month follow-up questionnaire to job trainers on participants' vocational performance. In-game performance: level score, time logs, surveys (modified version of Loewenthal's core elements of the gaming experience questionnaire). Participant survey: tiredness, immersion, motion sickness, satisfaction. Job trainer survey: effective training, reasonable design, accuracy.	Follow-up survey showed improvement in all of the trained skills. Most improvement was found in participants in the money management skills, cleaning, and social skills modules. Loading the back of a truck skill was found to be too difficult for individuals with high functioning ASD. Distractors did not produce negative effects on the performance of individuals with ASD.
Burke et al. (29) United States	ROBINS I: serious risk of bias	Virtual interactive training agents (ViTA): a VR job interview training platform using the Unity Game Engine, three cameras, Xbox Gen 1 Kinect camera to track facial expressions. Displayed on a television screen. Participants interacted with avatars, which asked 10–12 interview questions, with 144 different scenarios to choose from. Facilitated by program implementation team.	Single arm pre-post study. Persons with ASD and developmental disabilities *n* = 32 M = 23 (3.12). 21.88% female; 78.13% male	NIL	Twice per week over 14 weeks.	Vocational skills: job interviewing skills	Marino Interview Assessment Scale	A significant increase was found in final interview scores.
Burke et al. (30) United States	ROBINS I: moderate risk of bias	Virtual interactive training agents (ViTA): a VR job interview training platform accessed through a computer, with speakers, mouse, microphone, and keyboard. Participants interacted with avatars, which asked 10–12 interview questions, with 144 different scenarios to choose from. Facilitated by program implementation team, who worked with participants and teachers.	Within-subjects repeated measures design. Self-administered through a weblink. Majority of the participants had ASD, IDD, or ADHD/ADD and were in secondary or post-secondary educational institutions. *n* = 153 M = 21.71 (3.14). 26.76% female; 73.33% male	NIL	Approximately 22 weeks.	Vocational skills: job interview skills and self-efficacy	Job interview skills: Marino Interview Assessment Scale. Job interview self-efficacy: VITA-DMF Self Efficacy Scale	Significant improvements were found in interview skills and job interview self-efficacy.
Chang et al. (7) Taiwan	ROBINS I: critical risk of bias	ARCoach: a vocational task prompting system for food preparation training. AR tags were used to represent food items and the system provided sound alerts when food items were incorrect or misplaced. Facilitated by job coaches.	Pretest-posttest small sample study. Community-based supported employment service users with intellectual disabilities. *n* = 3 (21, 20, and 25 years)	NIL	One session per day for 5–6 days. A session consisted of a process of preparing five combo meals; each meal required four task steps.	Food preparation task	In-game performance: number of steps performed correctly at baseline, intervention and 4 weeks post-intervention (maintenance). NASA Task Load Index: to rate subjective perception of workload demand.	All three participants improved on their success rates, which were maintained. Participants indicated the mental and physical demands to operate the AR system were low.
Giachero et al. (31) Italy	ROBINS I: serious risk of bias	Semi-immersive VR scenarios (different steps in sowing seeds) were projected on a 50 inch curved screen for learning purpose. Group training was facilitated by therapist.	Single arm pretest-posttest design. Adults with ID in a residential home, IQ < 70. *n* = 14 M = 47.7	NIL	14 sessions of training with two training sessions per week, each lasting 1 h.	Gardening: ability to sow zucchini.	Self-developed questionnaire on steps required for sowing the zucchini. Other scales: Phonemic Fluency, Semantic Fluency, Frontal Assessment Battery, Attentive Matrices, Corsi Test, Spatial Supra-Span, Token Test, Verbal Span, Prose Memory Test, and MMSE.	Zucchini sowing questionnaire (carer): significantly greater percentage of correct responses after intervention. Zucchini sowing questionnaire (independent rater): significantly greater percentage of correct responses after intervention.
Humm et al. (32) United States	ROB2: high risk of bias	A virtual reality role-play utilizing PeopleSim^TM^ technology: Job Interview Training with Molly Porter was designed to teach, reinforce, and refresh job-interview skills. Trainers/facilitator: not stated.	Randomized controlled study: randomization at ratio of 1:2 (waitlist vs. experimental) Adults with ASD, schizophrenia/serious mental illness and veterans with PTSD *n* = 64 M = 50.0 (11.6)-schizophrenia M = 24.9 (6.7)-ASD	Waitlist control Adults with ASD, schizophrenia/ serious mental illness and veterans with PTSD *n* = 32 M = 44.3 (10.3)-schizophrenia. M = 23.2 (3.0)	10 h of training with Job Interview Training with Molly Porter (~20 trials) over the course of five visits in 2 weeks.	Job interview skills	Job interview skills: standardized interview role-plays and self-report of self-confidence; vocational data (no details provided). Neurocognitive and social cognitive assessments (RBANS and BLERT)	Role play scores: ANCOVA showed significant treatment condition effect for the Molly group. Self-confidence scores: ANCOVA showed significant training effect. Other results not reported.
Kuper et al. (33) United States	ROBINS I: serious risk of bias	Virtual reality instructions on how to wire an electrical outlet. Multimedia training stimuli (MTS) VR application was created and uploaded onto an Apple iPhone. The application was accessed through a Qualcomm Snapdragon 820 processor and by placing the phone into a cardboard VR viewer. Conducted by research team member in participants' natural settings (home, vocational assistance center, etc.).	Single arm pre-post study Community dwelling adults with autism. *n* = 10 Seven men and three women, ranging from 19 to 42 years of age.	NIL	Participants were watched a video on performing the electrical wiring task. After that, they took part in the VR portion of the MTS training. Duration of training not stated.	Vocational task: wiring an electrical outlet.	Modified version of the New General Self-Efficacy scale, qualitative feedback.	Participants showed a significant increase in their perceived self-efficacy to perform the task of wiring an electrical outlet. Participants also reported that they were engaged and had fun.
Smith et al. (34) United States	ROB 2: some concerns on risk of bias	Virtual reality job interview training (VR-JIT). A human resource manager character “Molly Porter” would ask questions about skills and experiences. Implemented on a computer monitor with speech recognition software. The character generated interview questions using an algorithm based on customizable features such as need for accommodations) and participants' responses. Conducted by two research staff members.	Randomized controlled study VR simulated interviews with three difficulty levels where Molly would be friendly (easy), business-oriented (medium), or brusque (hard). Adults with ASD *n* = 16 M = 24.9 (6.7)	Treatment as usual with no intervention. Adults with ASD *n* = 10 M = 23.2 (3.0)	Five training sessions, two h of working role play interviews lasting 20 min each time.	Vocational skills: job interviews	Vocational skills: role plays of job interviews using standardized actors; self-reported job interview confidence. Other measurements: RBANS, BLERT, Emotional Perspective-Taking Task, training experience questionnaire, in-game performance.	VR-JIT participants had greater improvement during live standardized job interview role-play performances and self-reported job interview confidence than control participants. VR-JIT simulation in-game performance scores increased over time. Participants attended 90% of lab-based training sessions and found VR-JIT easy-to-use, enjoyable, and they felt prepared for future interviews.
Smith et al. (35) United States	ROB2: some concerns in risk of bias	Virtual reality job interview training (VR-JIT). A human resource manager character “Molly Porter” would ask questions about skills and experiences. Implemented on a computer monitor with speech recognition software. The character generated interview questions using an algorithm based on customizable features such as need for accommodations) and participants' responses. Conducted by research staff members.	Randomized controlled study. VR-JIT delivered through educational content, interactive role-play simulator, and integrated feedback. Adults with ASD *n* = 15 M = 25.0 (6.9)	Treatment as usual with no intervention. Adults with ASD *n* = 8 M = 23.1 (3.3)	Six months follow-up period. Frequency and duration of intervention not mentioned.	Employment outcomes	Employment outcomes at 6-month follow-up: percentage of job interviews, offers, and job acceptances.	Logistic regression indicated VR-JIT participants had greater odds of attaining a competitive position than controls.
Smith et al. (36) United States	ROB 2: high risk of bias	Virtual reality job interview training (VR-JIT). A human resource manager character “Molly Porter” would ask questions about skills and experiences. Implemented on a computer monitor with speech recognition software. The character generated interview questions using an algorithm based on customizable features such as need for accommodations) and participants' responses. Conducted by research staff members.	Randomized controlled study. Randomization done on estimated ratio of 2 to 1 to optimize VR-JIT evaluation. VR-JIT delivered through educational content, interactive role-play simulator, and integrated feedback. Individuals with schizophrenia or schizoaffective disorder *n* = 21 M = 40.8 (12.2)	Treatment as usual with no intervention Individuals with schizophrenia or schizoaffective disorder *n* = 11 M = 39.1 (10.6)	10 h over five visits	Employment outcomes and vocational skills: job interview	Employment outcomes: job offers Job interview skills: role plays of job interviews using standardized actors.	Experimental group had greater odds of receiving a job offer by 6-month follow-up compared to controls (OR: 8.73, *p* = 0.04). More training was associated with shorter waiting time to job offer (*r* = −0.63, *p* < 0.001). Experimental group also demonstrated increased role-play scores between pre-test and post-test while controls did not (*p* = 0.001).
Smith et al. (37) United States	ROB 2: some concerns in risk of bias	Virtual Interview Training for Transition Age Youth (VIT-TAY), which was adapted from VR-JIT to cater to transition-age youths. Participants to apply for jobs in a fictional Company “Wondersmart” and interviewed by two virtual characters, with the help of virtual job coach. Adaptations included a token economy system, three additional learning goals across interview difficulty levels, screen reader capabilities, and social storytelling with the use of video and audio. Facilitated by teachers in Pre-employment transition Services (Pre-ETS).	Randomized controlled study: block randomized at a 2:1 ratio. Pre-employment transition Services (Pre-ETS) + VIT-TAY. Transition age youths with autism in high school special education settings *n* = 48 M = 19.8 (3.0)	Pre-employment transition Services (Pre-ETS) only: work-based learning experiences such as volunteering, job shadowing, and trial work experiences and workplace readiness training. Transition age youths with autism in high school special education settings. *n* = 23 M = 19.4 (2.6)	15 sessions (~45 min each)	Employment outcomes and vocational skills: job interview skills	Employment outcomes: employment at 6-month follow-up Job interview skills: single mock interview completed with a research team member using a version of the Mock Interview Rating Scale; self-reported job interview self-efficacy; self-reported job interview anxiety.	Logistic regression showed that Pre-ETS + VIT-TAY group was more likely to obtain competitive employment than the pre-ETS group Mock interview: significant group-by-time interaction for job interview skills total score improvement in Pre-ETS+ VIT-TAY TAY compared to Pre-ETS only. Significant reduction in job interview anxiety in experimental compared to control group.
Smith et al. (38) United States	ROBINS I: moderate risk of bias.	Virtual Interview Training for Transition Age Youth (VIT-TAY), which was adapted from VR-JIT to cater to transition-age youths. Participants to apply for jobs in a fictional company “Wondersmart” and interviewed by two virtual characters, with the help of virtual job coach. Adaptations included a token economy system, three additional learning goals across interview difficulty levels, screen reader capabilities, and social storytelling with the use of video and audio to reduce cognitive load. Facilitated by teachers in Pre-employment transition services (Pre-ETS).	Quasi-Experimental Hybrid Effectiveness-Implementation Study. VIT-TAY: Students with disabilities in 32 Pre-ETS schools, including ASD and ID. *n* = 356 M = 18.7 (2.2)	Processed data from the prior VR-JIT study were used for the evaluation. VR-JIT: Students with disabilities in 32 Pre-ETS schools, including ASD and ID. *n* = 279 M = 18.6 (1.5)	Approximately three 45- to 60-min VIT-TAY sessions per week over 4–6 weeks to achieve the recommended 15 virtual interviews.	Employment outcomes	Employment status after intervention and 6-month follow-up.	48.1% of participants using VIT-TAY obtained new jobs (*n* = 168) between baseline and follow-up, which was significantly higher than the 32.7% job attainment rate of students who used VR-JIT. 4.9% of VIT-TAY participants and 17.8% of VR-JIT participants who were employed at baseline, sustained their employment through follow-up. By follow-up, the unemployment rate was 47.0% in the VIT-TAY group and 49.5% in the VR-JIT group. 25.6% of the unemployed VIT-TAY participants obtained either a new paid or unpaid internship by follow-up, compared with 17.0% of VR-JIT students.
Sohn et al. (39) South Korea	ROBINS I: serious risk of bias	Virtual reality-based vocational rehabilitation training program (VR-VRTP) Practical situations commonly encountered in two types of occupations: convenience store employee and supermarket clerk. Participants spoke directly into a microphone and their voices were recorded, with a system in place for providing feedback. A three-dimensional surround screen using three digital projectors was installed in the immersion room. Patients used a mouse to move and respond to the virtual space. Facilitator: not stated.	Single arm pre-post study Outpatients with schizophrenia who were interested in vocational rehabilitation. *n* = 9 M = 36.65 (5.41)	NIL	Weekly over 8 weeks with the two scenarios. Both scenarios took around 35 min.	Vocational skills	Personal and social functioning: PSP. Cognitive scales: WCST, Stroop Test, RCFT, and Korean Version of the of the Auditory Verbal Learning Test (K-AVLT). Clinical scales: Manchester Scale, Clinical Global Impression, Hamilton Depression Rating Scale, Zung Depression Rating Scale, BAI.	Significant improvement in PSP scores. Significant improvement also observed in general symptoms on the Manchester Scale, K-AVLT, and delayed recall on the RCFT. No statistically significant change of WCST and Stroop Test scores. However, the memory scores on both the immediate recall and delayed recall portions of the RCFT and on the first and fifth attempts of the K-AVLT increased significantly.
Strickland et al. (40) United States	ROB 2: some concerns in risk of bias.	JobTIPS: web-based education and a virtual reality job interview training delivered on Venugen4 platform. Clinician assumed the avatar role of “interviewer” remotely and the treatment group participant assumed the role of “interviewee.” Participant was supervised by a staff in a university research facility and engaged with the clinician in dialogue *via* headphones and speakers.	Randomized controlled study Experimental group received JobTIPS, which consisted of Theory of Mind-based guidance, video models, visual supports, and virtual reality practice sessions in teaching appropriate job interview skills. Youths with ASD *n* = 11 M = 18.21 (1.03)	Control group did not receive intervention. Youths with ASD *n* = 11 M = 17.66 (1.27)	Participants completed web-based contents consisting of seven subsections and one 30-min virtual practice session with a clinician.	Vocational skills: job interview. Social and communication skills.	Simulated interview using self-developed Interview Skills Rating Instrument with two sub-scales: Response Content and Response Delivery. Social and communication skills: SRS.	Treatment group showed a significant positive change at the second interview on the Response Content. No significant differences in SRS scores between experimental and control group. None of the SRS scores correlated significantly with outcomes on the Interview Skills Rating Instrument.
Tsang and Man (41) Hong Kong	ROB 2: low risk of bias.	Virtual Reality-based Vocational Training System (VRVTS): a VR boutique scenario (3D non-immersive), delivered on a computer displayed on a wide LCD monitor with stereo speakers. Content divided into three levels, based on salesperson training that required social skills, flexibility and problem solving. Participants interacted with joystick and keyboard. Led by therapist.	Randomized controlled study. Participants in all three groups received similar 3-h prevocational skills training in work-simulated workshops in the Occupational Therapy Department. Vocational Rehabilitation Group (VRG): apart from the prevocational skills training, participants were required to attend VRVTS. Inpatients with schizophrenia receiving vocational rehabilitation services in a psychiatric hospital. *n* = 25 M = 39.60 (7.96)	Two control groups: Therapist-Administered Group (TAG): Training done according to the training manual with contents similar VRG. Convention Group (CG): 3-h daily prevocational skills training in work-simulated workshops similar to the other two groups but no added intervention. **TAG** *n* = 25 M = 40.76 (9.19) **CG** *n* = 25 M = 41.56 (9.94)	At least 3 h of prevocational skills training in every working day during hospitalization. VRG/TAG 10 sessions of training over 5 weeks. Each session lasted for about 30 min.	Vocational skills.	Vocational skills: VCRS, self-developed on-site test on participants' knowledge on sales, self-developed participants' report on self-efficacy in sales-related activities. Other scales: Brief Neuropsychological Cognitive Examination, Digit Vigilance Test, Rivermead Behavioral Memory Test, and WCST.	Marginally significant interaction effect of group over time found for VCRS. Significant interaction effect of group over time in on-site test. Both the VRG and the TAG showed significantly better improvements in the on-site test than the CG. No significant interaction effect of group over time in self-efficacy scores. *Post-hoc* comparison found that only the VRG showed a significantly better improvement in the self-efficacy score than the CG. VRG showed a better performance than both the TAG and the CG in the WCST.

**Table 3:** Studies that addressed social skills and social participation.

**Reference and country**	**Assessment of bias (ROB 2/ROBINS-I)**	**Treatment modalities/methods and therapist/trainer facilitation**	**Experimental condition^a^**	**Control condition (if applicable)^b^**	**Duration and frequency of intervention**	**Skills and/or functional domains targeted**	**Outcome measurements**	**Results/effects of intervention**
Adery et al. (42) United States	ROBINS I: serious risk of bias	Multimodal Adaptive Social Intervention in Virtual Reality (MASI-VR): administered on a desktop computer in the form of a video game. Participants engaged with avatars to learn skills on starting a conversation. Conducted in a research laboratory by research team.	Single arm pretest-posttest design. Community individuals with a diagnosis of schizophrenia. *n* = 16 M = 48.63 (6.98)	NIL	Ten sessions (twice a week). Every session required participant to complete 12 social “missions.”	Social engagement and functioning	Social functioning: SFS Other scales: BPRS, SANS, Scale for the Assessment of Positive Symptoms, National Adult Reading Test Revised, Wechsler Abbreviated Scale of Intelligence.	No significant changes overall or across subscales of the SFS. Psychiatric symptoms: medium-to-large effect sizes in symptom severity reduction on BPRS and SANS.
Amaral et al. (43) Portugal	ROBINS I: serious risk of bias	VR-assisted Brain-Computer-Interface (BCI) training for social attention: VR Oculus Rift DK 2 headset with eye movement tracking embedded with Eye Tracking HMD. EEG cap with 16 electrodes was worn by participants as a BCI device. Participants watched animations in four virtual scenarios. Facilitator: research team.	Single arm pretest-posttest design. Participants with autism spectrum disorder (ASD) *n* = 15 M = 22.2 (5.5)	NIL	Seven sessions over 4 months.	Adaptive functioning and social attention.	Adaptive functioning: VABS. Social attention: eye fixations. Other scales: Autism Treatment Evaluation Checklist, Profile of Mood States, HADS, and BDI.	Evident improvement in Adapted Behavior Composite of VABS, reduction in depression, joint attention did not show changes.
Jacques et al. (44) Canada	ROBINS I: critical risk of bias.	Decoding Social Interaction Task in VR (DSIT in VR): a psychoeducational program on decoding social cues conducted in Psyche, a fully immersive stereoscopic and wireless 6-wall CAVE-Like system where participants moved freely with a wand tracker and were immersed in five socials contexts. Facilitated by research member with clinical experience.	Single arm pre-post study *n* = 3 Three adult males with ASD, typical intelligence.	NIL	Not described	Social skills and social cue perception.	Social Interaction Self-Statement (SISST): to assess participants' positive and negative thoughts about their social experiences. Rating grid of behaviors involved detecting social cues (CPS); perception of social decoding and social skills (CPI). CPS and CPI are in-house tools, no further elaboration given.	SISST-P: three reported improvements in positive thoughts. SSIST-N: one reported reduction in negative thoughts. CPS (parents-rated): two out of three reported. improvements in social skills. CPS (participants rated): none reported improvement in social skills. CPI (parent rated):1 reported reduction of maladaptive behavior. CPI (participants rated): two reported reduction of maladaptive behavior.
Kandalaft et al. (45) United States	ROBINS I: serious risk of bias	Virtual Reality Social Cognition Training (VR-SCT) intervention. The virtual reality environment included many locations such as an office building, a pool hall, a fast-food restaurant, a technology store and an apartment. Avatars (displayed on computers and controlled by keyboard and mouse) were created to look like coach clinicians and participants. Facilitated by two clinicians: the “coach” and the “confederate.”	Small sample single-arm pretest-posttest design *n* = 8 (18–26 years). All were diagnosed with either Asperger Syndrome or PDD-NOS.	NIL	Ten sessions, 2 per week, 1 h each.	Social skills performance and social cognition (emotion recognition and theory of mind)	Social performance: Social Skills Performance Assessment Emotional affect recognition: ACS-SP, Facial Expressions of Emotion Stimuli and Tests, Ekman 60. Theory of Mind: Reading the Mind in the Eyes, Social Perception Task. VR-SCT Follow-up Survey: self-developed survey to assess the long-term impact of the VR-SCT on social skills, cognition and functioning.	Significant improvement in social skills performance, theory of mind and emotion recognition.
Kumazaki et al. (46) Japan	ROB2: high risk of bias	Autism-Focused Public Speech Training using Simple Virtual Audiences (APSV): participants did public speaking in front of the system with agent's eye movement and nodding behavior in response to participant's performance. The subsequent trial procedures were conducted from Days 1 to 7. Facilitated by research team.	Randomized controlled study. Mock public speaking experience in front of the APSV system. Individuals with ASD *n* = 8 male M = 23.6 (5.3)	Independent Study (IS): participants in IS group read and addressed the same public speaking material in an empty room (matched in terms of duration and frequency of APSV sessions). *n* = 7 M = 24.7 (4.9)	Daily for 10 min over 7 days.	Public speaking	Functional outcomes: mock public speaking in front of 10 people, followed by questionnaire on self-confidence during public speaking. Other measurements: Liebowitz Social Anxiety Scale, Attention Deficit Hyperactivity Disorder Rating Scale, salivary cortisol level.	Self-confidence improved and salivary cortisol levels were significantly decreased in the APSV group as compared to those in the IS group.
Pot-Kolder et al. (47) Netherlands	ROB2: some concerns on risk of bias.	Virtual-Reality Based Cognitive Behavior Therapy (VR-CBT) Four virtual social environments (a street, bus, café, and supermarket) were created with Vizard software. Participants used head-mounted display. Conducted by psychologists with CBT training.	Randomized controlled trial. VR-CBT and individualized case formulation guided exposure. Outpatients. Majority diagnosed with Schizophrenia. *n* = 58 M = 36.5 (10). More than half were males.	Treatment as usual antipsychotic medication, regular contact with psychiatrist and psychiatric nurse. Outpatients. Majority diagnosed with Schizophrenia. *n* = 58 M = 39.5 (10) More than half were males.	Sixteen sessions over 8–12 weeks. Sessions lasted 1 h (40 min VR games and 20-min reflection).	Social participation (objective and subjective)	Social participation: ESM (PsyMate), Social functioning: SOFA, Manchester Short Assessment of Quality of Life. Other scales: Igroup Presence Questionnaire, Simulator Sickness Questionnaire, Safety Behavior Questionnaire Persecutory Delusions, Green et al. Paranoid Thoughts Scale, Social Interaction Anxiety Scale, BDI, Internalized Stigma of Mental Illness, Brief Core Schema Scales, Davos Assessment of Cognitive Biases Scale, Brief Adherence Rating Scale.	Treatment effect was found on time spent with others (objective social participation) at follow-up (6 months after baseline) compared with baseline. VR-CBT group had improvements in self-stigmatization and social functioning at follow-up. No significant differences in QOL. Large reduction in momentary paranoia and anxiety.
Rus-Calafell et al. (48) Spain	ROBINSI: serious risk of bias	VR integrated program as an adjunct to a brief social skills intervention for participants with schizophrenia. A laptop with a 15.6-inch monitor and stereoscopic view was used. Participants were required to use 3D glasses and headphones. Conducted by clinical psychologist (first author).	Repeated measures single arm design. Soskitrain program: participants practiced social interactions with virtual avatars with facial expressions, which encouraged progressive learning of the social skills and provided positive or negative reinforcement. *n* = 12 M = 36.50 (6.01)	NIL	Sixteen individual 1-h sessions conducted twice a week over 8 weeks.	Social functioning and social skills	Social functioning: SFS. Social performance: Assertion Inventory; Simulated Social Interaction Test; Social Avoidance and Distress Scale. Other scales: PANNS.	Repeated measures ANOVA showed significant improvements in social functioning, social anxiety and discomfort, avoidance and negative symptoms. Objective scores showed a pattern of learning in emotion perception, assertive behaviors and time spent in a conversation, with gains maintained at 4-month follow-up.
Vass et al. (49) Hungary	ROB 2: low risk of bias.	VR-based Theory of Mind Intervention (VR-ToMIS): simulated social interactions with an avatar in immersive VR environments, with virtual conversations dialogue to train Theory of Mind. Head-mounted display (HMD), a Samsung S7 smartphone and a Samsung Simple Controller were used. Conducted by a psychotherapist.	Randomized controlled, single-blind study VR-based Theory of Mind intervention (VR-ToMIS)—structured and individualized method using cognitive and behavioral therapeutic techniques. Outpatients with schizophrenia. *n* = 9 M = 38.6 (13.49)	Control group: passive VR using the same software but without intervention. Participants freely explored virtual destinations but could not contact avatar. Outpatients with schizophrenia. *n* = 8 M = 48.8 (8.87)	9 weeks 50 min per session per week in individual setting.	Social participation and social cognition.	Quality of life: Lancashire Quality of Life Profile. Pragmatic language skills: Hungarian metaphor and irony test. Other scales: RBANS, WCST-64, Baron-Cohen Mind in the Eyes Test. Structured interviews with participants and their relatives.	No significant change in quality of life. Pragmatic language skills: significant between-group differences were followed by large effect size observed in metaphor. Visuospatial and attention subtest of RBANS significantly differed between groups, with medium effect size favoring VR-ToMIS group. Significant between-group differences in the first- and third-order theory of mind tasks in favor of the VR-ToMIS group.
White et al. (50) United States	ROB 2: some concerns in risk of bias.	Brain-Computer Interface for ASD (BCI-ASD): A VR-based BCI platform where participants interacted with avatars in social situations and social activities. The BCI headband provided feedback (visual or textual) on participants' attention levels. The intervention was administered on a desktop computer and a tablet in a clinical office setting. The software allowed deployment in an immersive environment. Facilitator: not stated.	Small randomized study. BCI-ASD group. Adults with ASD attending college with at least one co-occurring diagnosis. *n* = 4 M = 20.75 (1.71)	Control group: College for Living Success (CLS): A psychosocial and support program grounded in CBT and mindfulness-acceptance based approaches. Facilitated by therapist. Adults with ASD attending college with at least one co-occurring diagnosis. n = 4, mean age = 20.25 (1.71)	BCI-ASD: 10–14 weekly sessions, each lasting between 15 and 30-min. CLS: 2 h per week.	Social participation and adaptive functioning	Social participation and adaptive functioning: Clinical Global Impression-Improvement, College Living Experience Satisfaction Scale, Student Adaptation to College Questionnaire Other scales: Barkley Deficits in Executive Functioning Scale	No clinically meaningful change on overall adaptation to college, academic adjustment, attachment, personal-emotional adjustment, and social adjustment for any participant. 2 CLS participants and 2 BCI-ASD participants showed significant decline in overall adaptation on the Student Adaptation to College Questionnaire. 1 BCI-ASD participant demonstrated significant improvement in overall adaptation to college during the intervention period. 2 BCI-ASD participants showed significant decline in academic adjustment. 1 CLS and 1 BCI-ASD participant showed significant decline on personal-emotional adjustment. 1 BCI-ASD participant showed significant improvement in social adjustment, 1 CLS and 2 BCI-ASD participants showed significant decline. Overall, no significant improvement in executive functioning from pre- to post- for either intervention.

**Table 4:** Studies that addressed quality of life.

**Reference and country**	**Assessment of bias (ROB 2/ROBINS-I)**	**Treatment modalities/methods and therapist/trainer facilitation**	**Experimental condition^a^**	**Control condition (if applicable)^b^**	**Duration and frequency of intervention**	**Skills and/or functional domains targeted**	**Outcome measurements**	**Results/effects of intervention**
Dellazizzo et al. (51) Canada	ROBINS I: serious risk of bias	Virtual reality assisted therapy (VRT): participants created an avatar best resembling the most distressing entity believed to be the source of the malevolent voice and engaged in a dialogue with it. Idiosyncratic avatars were created using Unity 3D game engine and voice was simulated with a voice transformer. Participants were immersed through Samsung GearVR head-mounted display and smartphone. Facilitated by a psychiatrist. Participants engaged with the therapist *via* the personalized avatars.	Single arm pretest-post-test. Participants who had completed Cognitive-Behavioral Therapy for Auditory Verbal Hallucinations received VRT. Persons with refractory schizophrenia or schizoaffective disorders. *n* = 10 M = 43.4 (no SD provided). 20% female and 80% male.	NIL	Six sessions 1 h per session	Quality of life	Quality of life: Quality of Life Enjoyment and Satisfaction Questionnaire-Short Form (QLESQ-SF). Other scales: Psychotic Symptoms Rating Scale, Beliefs About Voices Questionnaire-Revised, BDI-II, and PANNS.	Significant changes from pre-VRT to follow-up VRT, yielding a moderate effect size.
Du Sert et al. (48) Canada	ROB2: high risk of bias	VR-assisted therapy (VRT): Immersive virtual reality in which participants created an avatar best resembling the most distressing entity believed to be the source of the malevolent voice and engaged in a dialogue with it. Idiosyncratic avatars were created using Unity 3D game engine and voice was simulated with a voice transformer. Participants were immersed through Samsung GearVR head mounted display and smartphone. Facilitated by a psychiatrist. Participants engaged with the therapist *via* the personalized avatars.	Randomized, partial cross-over trial. VR-assisted therapy (VRT) group Persons with refractory schizophrenia or schizoaffective disorders. Total *n* = 15 (unclear on sample size in experimental and control conditions, although randomized in 1:1 ratio) M = 42.9 (total participants including controls)	Treatment-as-usual.	7 weekly sessions (one avatar creation session and six 45-min therapeutic sessions).	Quality of life.	Quality of life: Quality of Life Enjoyment and Satisfaction Questionnaire-Short Form (QLESQ-SF) Other scales: Psychotic Symptoms Rating Scale, Beliefs About Voices Questionnaire-Re-vised, PANNS, and BDI-II	No analysis between experimental and control group reported. Statistically significant change in QLESQ-SF scores between baseline and post-intervention.
Maskey et al. (52) United Kingdom	ROBINS-I: serious risk of bias	Cognitive Behavior Therapy combined with Virtual Reality Exposure (VRE) to reduce anxiety. Initial sessions conducted with participants and their supporters at home. Subsequently, the participants and supporters visited Blue Room VRE, a 360-degree seamless screened room with computer-generated images projected onto the walls and ceilings. No headsets and goggles used and navigated using tablet by psychologist. Conducted by clinical psychologist.	Single arm pre-post study Adults diagnosed with ASD. *n* = 8 M = 29.8 (range 18.8–57.0)	NIL	One session with therapist to learn anxiety management techniques. Then, four 20-min sessions of graded exposure in an immersive VR room. Participant then tried real-life exposure. Measured progress at 6 weeks and 6 months after the last VR session.	Quality of life and self-reported functional outcomes	Quality of life: WHOQOL-BREF Self-report ratings of participants' confidence in managing the target anxiety situation, using 6-point visual analog scale. Other scales: BAI, GAD-7, and PHQ-9.	No significant changes in WHOQOL-BREF measures. Five out of eight participants reported improvements in day-to-day situations.

^a^Sample characteristic: n = x denotes the number of patients in that condition, M = mean age in years and standard deviation (given in parentheses), sample type.

^b^Sample characteristics: n = x denotes the number of patients in that condition, M = mean age in years and standard deviation (given in parentheses), sample type.

ACS-SP, Advanced Clinical Solutions for WAIS-IV and WMS-IV Social Perception Subtest; BAI, Beck Anxiety Inventory; BDI, Beck Depression Inventory; BDI-II, Beck Depression Inventory II; BLERT, Bell-Lysaker Emotion Recognition Task; BPRS, Brief Psychiatric Rating Scale; ESM, Experience Sampling Method; GAD-7, Generalized Anxiety Disorder 7; HADS, Hospital Anxiety and Depression Scale; HMD, Head Mounted Display; IQ, Intellectual Quotient; MBI, Modified Barthel Index; MMSE, Mini Mental State Examination; MoCA, Montreal Cognitive Assessment; PDD-NOS, Pervasive Developmental Disorder-Not Otherwise Specified; PHQ-9, Patient Health Questionnaire-9; PSP, Personal and Social Performance Scale; PTSD, Post-Traumatic Stress Disorder; QOL, Quality of Life; RBANS, Repeatable Battery for the Assessment of Neuropsychological Status; RCFT, Rey–Osterrieth Complex Figure Test; SANS, Scale for the Assessment of Negative Symptoms; SOFA, Social and Occupational Functioning Assessment Scale; SRS, Social Responsiveness Scale; SFS, Social Functioning Scale; VABS, Vineland Adaptive Behavior Scales; WAIS III, Wechsler Adult Intelligence Scale III; VCRS, Vocational Cognitive Rating Scale; WCST, Wisconsin Card Sorting Test; WHOQOL-BREF, World Health Organization Quality of Life abbreviated.

### The deployment of technology

#### Augmented reality (AR)

Out of the 38 selected studies for review, only three studies used AR in their interventions (7, 19, 23). One study developed independent living tasks videos (ironing, bed making, and setting an alarm clock) and presented them on an AR application installed on an iPad (19). As the study was conducted in a university campus dormitory for adults with ID, laminated targets were set up in the participants' rooms or common areas. During intervention, participants pointed the iPad cameras at laminated targets to activate video models, which appeared as an overlay across the target. This facilitated the learning of daily living skills within their natural environment.

Another study used AR to teach the self-care skill of tooth brushing (23). The three-dimensional motion sensor in the smart toothbrush enabled recognition of the participants' hand movements and provided a visual guide for posture and direction. This form of instructional guidance using visual feedback allowed participants to learn in a more interactive manner.

The third study also used AR as training feedback. In this intervention named ARCoach, AR was used as a vocational task prompting system for food preparation training (7). AR tags were used to represent food items and the system provided sound alerts when participants made errors in the steps of the tasks.

#### Virtual reality (VR) with head-mounted display (HMD)

The studies utilizing VR also harnessed the technology differently. Six studies immersed the participants in the virtual environment using a head-mounted display (HMD). Bozgeyikli et al. (28) used a VR-based vocational training platform, to train six transferrable vocational skills. Three of the six skill modules (cleaning, shelving, and environmental awareness) were designed to be performed while wearing the HMD while the remaining three skill modules (loading the back of a truck, money management, and social skills) were presented on a 180° curved screen. Facilitation by job trainers were done using a remote-control interface on a separate tablet. Another study by Simões et al. (27) used a VR Oculus Rift headset for participants to experience the full immersion of using public bus transportation. Their anxiety levels were also monitored by a bracelet that captured electrodermal activity.

Three studies used immersive VR to assist in cognitive behavior therapy for persons with schizophrenia, in order to improve their social participation and quality of life. Participants in the Virtual-Reality Based Cognitive Behavior Therapy (VR-CBT) were given guided exposure to four virtual social environments (a street, bus, café, and supermarket) by viewing them through HMDs (47). On the other hand, participants in VR-assisted Therapy (VRT) created an avatar that resembled the source of their auditory hallucinations and engaged in a dialogue with it through Samsung Gear HMD and smartphone (48, 51).

Lastly, one study used HMD to immerse participants in simulated social interactions with an avatar to train theory of mind (53). A smartphone and handheld controller were used alongside to facilitate virtual conversations.

#### Virtual reality (VR) with cardboard viewer or 3D glasses

In two studies, a VR application was installed on a smartphone and slotted into the Cardboard Viewer. Kuper et al. (33) used this technology to train participants on the skill of wiring an electrical outlet, while Miller et al. (24) simulated the whole process of air travel, from entering the virtual airport, checking in, navigating security, waiting at the departure gate, and boarding the airplane.

On the other hand, the Soskitrain program by Rus-Calafell et al. used 3D glasses and headphones in a brief social skills intervention for participants who had schizophrenia (54). A laptop and a 15.6-inch monitor with stereoscopic view was also used. Participants practiced social interactions with virtual avatars who used their facial expressions to provide feedback. This feature encouraged progressive learning of social skills through positive or negative reinforcement.

#### Virtual reality (VR) on desktops or laptops

Almost half of the studies (18 studies) deployed VR on desktops or laptops and participants used a keyboard or mouse/joystick to interact within the virtual scenarios. Microphones and headphones were also used in interventions that involved verbal interactions. Several of these studies made use of virtual characters, speech recognition software and artificial intelligence algorithm to create an interactive platform to train skills such as job interviewing and public speaking. In Virtual Reality Job Interview Training (VR-JIT) and Job Interview Training with Molly Porter programs, the human resource manager “Molly Porter” generated interview questions based on the participants' job modification needs and their verbal responses (32, 34–36). A subsequent adapted version added screen reader capabilities and a virtual job coach that provided additional support and feedback (37, 38).

Other studies presented non-immersive VR simulated cities, towns or workplaces on desktops/laptops with large screens, so that participants could learn navigation, vocational skills, community living skills, public speaking or social skills by using keyboard or joysticks (17, 20, 22, 41, 42, 45, 46).

#### Virtual reality (VR) with other features

Two studies added Brain-Computer-Interface (BCI) features by using electroencephalogram (EEG) cap or headband. Participants in a social attention training program put on an Oculus Rift headset with eye tracking package to watch four animated scenarios and their attention was tracked using an EEG cap with 16 electrodes (43) Participants in another study interacted with avatars in social situations and their attention levels were tracked by a BCI headband, which provided visual or textual feedback on their attention levels (50).

One study added Kinect as a motion capture device, which enabled movements to be mapped onto the virtual space. Participants who were learning navigation and street crossing could see their full-body movements mapped onto the VR city, which enabled them to have an authentic sense of their body within the three-dimensional virtual environment (26).

Two studies used Cave Automatic Virtual Environment (CAVE) rooms to maximize the immersion experience. The first study coined its room as the “Blue Room VR Exposure,” which was a 360-degree seamless screened room with computer-generated images projected onto the walls and ceilings (52). Participants diagnosed with ASD were gradually exposed to their phobic situation (such as getting onto a bus or going into a shop) within this immersive environment without the use of headsets or goggles. Navigation was controlled by a tablet. The second study used a similar fully immersive stereoscopic and wireless six-wall CAVE-like system where participants with ASD moved freely with a wand tracker (44).

Two studies utilized VR driving simulators to train driving skills and executive functioning for adults with ASD (18, 21). Lastly, a study reported on the use of a VR application installed onto tablets to train community living skills such as shopping at a supermarket and preparing a suitcase (25). The program was self-administered at home with caregivers, with a chat tool to contact a day rehabilitation staff member if assistance was required.

### Functional domains addressed

The functional domains which were addressed included self-care/community living, employment and social participation. The outcome measurements ranged from in-game performance of functional skills to real-world functional outcomes such as employment rates. Several studies also used observer-ratings of functional performance such as the Personal and Social Performance (PSP) scale and Modified Barthel Index (MBI). In addition, a few studies measured subjective quality of life.

#### Self-care and community living

Eleven studies reported interventions that addressed self-care and community living. Out of these studies, four of them created a simulated town or facility to train community mobility and navigation. In one study, a VR intervention was conducted by a therapist weekly over 10 sessions to train outpatients with ASD in navigation and street crossing (26). In-game street crossing performance was measured within the VR application. Although the study found no significant reduction in street-crossing errors, navigation became more efficient. In addition, caregivers reported a significant post-treatment improvement in the participants' real-world navigation performance. Another study used VR to train adults with ASD in taking public bus transportation (27). Their anxiety was tracked using electrodermal activity recording throughout the three intervention sessions. In-game measurement on action accuracy (number of correct steps) and post-game debriefing accuracy (getting participants to describe each step of the task) were used. At the end of the intervention, there was a statistically significant increase in debriefing accuracy and a significant reduction in anxiety among the participants. In another study, de la Torre-Luque et al. (22) created a VR environment of a disability center and engaged its service users with ID in daily navigation training of its ground floor facilities over 15 sessions. At post-intervention, the participants were able to perform real-life location tasks with a significant reduction in errors and time taken. Lastly, one small study trained adults with ASD in air travel by creating a virtual airport (24). Speech and language therapists facilitated the weekly 20-min sessions over 3 weeks by training participants on the use of functional language skills, which helped them interact and navigate busy environments such as an airport. Participants showed improvements in functional language skills and were able to accurately describe the sequence of air travel (24).

Two studies targeted the functional skills of driving in adults with ASD using VR driving simulators. The Cognitive Behavioral Intervention for Driving (CBID) consisted of cognitive behavioral strategies to enhance executive functions and generalizing them to driving (18). Intervention was carried out weekly over 10 sessions. Among the 19 participants who completed the intervention, eight obtained a driver's permit and one obtained a driver's license within 2 months. Another study on Virtual Reality Driving Simulation Training (VRDST) explored if outcomes could be different with automated feedback mechanism or eye-tracking device (21). Participants in the Automated VRDST group received real-time feedback *via* the simulator's computerized voice, instead of being coached by trainers in Standard VRDST. On the other hand, participants in the Eye-Tracking VRDST group wore eye-tracker glasses during driving, from which videos of their eye gazes were produced for the trainer's review and feedback. Results of tactical simulator tests showed no significant differences across the three VRDST groups (21).

The studies that trained participants in Activities of Daily Living (ADLs) were mainly implemented in the participants' natural settings. A study by Panerai et al. (25) found that caregivers of persons with ID could utilize a VR application on tablets at home, to train persons with ID in community living skills such as shopping at a supermarket and preparing a suitcase. *In-vivo* tests of these daily living tasks showed an increase in the number of correct responses and a reduction in prompts and number of errors. High levels of participant and family satisfaction were also reported (25). Another small-scale study using AR for three adults with IDD living in a school dormitory found that their independent living skills (e.g., ironing, bed making, and setting an alarm clock) improved immediately after intervention (19). Finally, a quasi-experimental study on adults with ID in residential facilities found that compared to traditional tooth brushing training, an AR-facilitated tooth brushing intervention yielded significantly better improvement in Modified Barthel Index (Korean version) scores and Simplified Oral Hygiene Index (23). This intervention was conducted twice a week for 12 weeks by an occupational therapist.

Two studies incorporated cognitive rehabilitation into community living skills training. In Câmara et al.s' single-blind randomized controlled trial of the Reh@City v2.0 intervention, participants with schizophrenia engaged in cognitive training through performing ADLs such as baking cookies at home and buying groceries in the supermarket (20). This was conducted as part of an inpatient psychosocial rehabilitation program. Participants in the comparison Task Generator group engaged in paper and pencil tasks. After 2 months of intervention, the Reh@City v2.0 group did not show significant improvement in quality of life but had significant improvements in overall cognition and immediate recall (20). Another study by Amado et al. (17) used a VR simulated town where outpatients with chronic schizophrenia or schizoaffective disorders were trained by a psychologist and occupational therapist to plan their actions around the town. After 12 interactive 90-min weekly sessions, participants showed significant improvements in Social Autonomy Scale total scores. In addition, there were significant improvements in attention and working memory but not executive functioning (17).

#### Vocational skills and employment

There were 15 studies which utilized VR or AR to provide vocational skills training or to enhance employment outcomes. Nine out of these 15 studies focused on job interview training. Smith and colleagues implemented the Virtual Reality Job Interview Training (VR-JIT), where a virtual human resource manager “Molly Porter” would ask questions and adopt different demeanor based on an algorithm of difficulty levels and customizable features (34). In a randomized controlled study of adults with ASD, VR-JIT participants demonstrated greater improvement during live standardized job interview role-play performances and self-reported job interview confidence than control participants (34). Participants attended 90% of the five-session lab-based training and found VR-JIT easy to use and enjoyable. A 6-month follow-up study of these participants showed that VR-JIT participants had greater odds of attaining competitive jobs than controls (35). The same intervention also yielded similar benefits for individuals with schizophrenia or schizoaffective disorder (36). Compared to treatment-as-usual, participants who underwent VR-JIT demonstrated better role-play performance and had greater odds of receiving a job offer by 6-month follow-up. More training was associated with shorter waiting time to job offers (36).

Smith and colleagues subsequently adapted VR-JIT and developed the Virtual Interview Training for Transition Age Youth (VIT-TAY), to cater to youths with ASD attending pre-employment transition services (Pre-ETS) in schools (37). Adaptations included a token economy system, three additional learning goals across interview difficulty levels, screen reader capabilities, and social storytelling with the use of video and audio to reduce cognitive load. The VIT-TAY was facilitated by teachers in the high school special education settings delivering Pre-ETS. After 15 sessions of VIT-TAY, participants showed better job interview skills (as measured by Mock Interview Rating Scale) and significant reduction in job interview anxiety than those receiving Pre-ETS only (37). A logistic regression also showed that the VIT-TAY+Pre-ETS group was more likely to obtain competitive employment after 6 months than the Pre-ETS only group (37). A subsequent study comparing VIT-TAY outcomes with processed data from the prior VR-JIT study found that 48.1% of VIT-TAY participants obtained new jobs (*n* = 168) between baseline and follow-up, which was significantly higher than the 32.7% job attainment rate of VR-JIT participants (38).

Another job interview VR program was the Virtual Interactive Training Agents (ViTA) by Burke et al. (29). In this program, participants with ASD or ID interacted with avatars, which asked 10–12 interview questions, with 144 different scenarios to choose from. This intervention was facilitated by the program implementation team, who worked with the participants and their teachers. A pilot single-arm study found significant improvement in Marino Interview Assessment Scale scores after intervention (29). A subsequent larger study was conducted using within-subjects repeated measures design and included participants with ASD, ID, and ADHD (30). Results showed that ViTA participants had significant improvements in job interview self-efficacy and job interview skills after intervention (30).

Other job interview training programs included JobTIPS, where the clinician assumed the avatar role of an “interviewer” remotely and the intervention group participants with ASD were coached by program staff in theory of mind and given visual supports to demonstrate appropriate job interview skills (40). There were no significant differences in Social Responsiveness Scale scores between JobTIPS and control participants, although JobTIPS participants performed better in aspects of job interview skills (40). Another study used the “Molly Potter” software with both adults with schizophrenia and adults with ASD. There were significant treatment condition effects for the Molly Porter intervention group when compared to waitlist controls, in terms of performance in standardized interview role-plays and self-confidence (32).

There were six studies which used VR or AR to train vocational skills relevant to open or sheltered employment in a vocational rehabilitation program. The Virtual Reality-based Vocational Training System (VRVTS) provided a non-immersive VR boutique environment to train inpatients with schizophrenia receiving vocational rehabilitation services in a psychiatric hospital (41). Participants were trained in vocational skills required of a salesperson, such as work-related social skills, flexibility, and problem solving. Participants randomized to VRVTS reported significantly better self-efficacy in sales-related activities than the control group (41). Another study on the Virtual Reality-based Vocational Rehabilitation Training Program (VR-VRTP) presented virtual work scenarios encountered by a convenience store employee and a supermarket clerk (39). Participants spoke directly into a microphone and their voices were recorded, with a system in place for providing feedback. In this small-scale single-arm pre-post study, outpatients with schizophrenia showed significant improvement in Personal and Social Performance scale scores after 8 weeks of VR-VRTP (39). A study by Bozgeyikli et al. on “Virtual Reality System for Vocational Rehabilitation of Individuals with Disabilities” (VR4VR) employed a closed-loop adaptive VR-based vocational training platform to train six transferrable vocational skills of cleaning, shelving, environmental awareness, loading the back of a truck, money management, and social skills (28). In this quasi-experimental small sample study involving adults with ASD and neurotypical individuals, job trainers reported improvements by ASD participants in all the trained skills, especially in money management, cleaning, and social skills (28).

Three of these six studies utilized technology to train specific vocational tasks. One study used VR to train community-dwelling adults how to wire an electrical outlet in their vocational assistance center or at home (33), while another study used AR as a task prompting system to aid job coaches in training food preparation for community-based supported employment service users with ID (7). Lastly, semi-immersive VR scenarios were used to teach adults with ID in a residential home the work task of sowing zucchini seeds (31). These three studies reported that participants were able to learn the specific tasks well, although no validated measurements were used (7, 31, 33).

#### Social skills and social participation

Nine studies utilized VR to train social skills or to facilitate social participation. Majority of these studies involved getting participants to interact with virtual avatars in different social scenarios, with feedback provided by the VR platform. For example, a study by Rus-Calafell et al. adopted a VR software called Soskitrain as an adjunct to a brief social skills intervention for participants with schizophrenia (54). The clinical psychologist coached participants to work on the feedback given by virtual avatars based on their facial expressions. At the end of the 16 individual 1-h sessions, participants showed significant improvements in social functioning, social anxiety, avoidance and negative symptoms, with majority of the gains maintained at 4-month follow-up (54). Another study required participants with schizophrenia to complete 12 virtual social missions over a 5-week program (42). Although reduction in psychiatric symptoms were seen, there was no significant change in Social Functioning Scale scores (42).

Three studies used VR to train aspects of social cognition. The Virtual Reality Social Cognition Training (VR-SCT) intervention used different virtual environments and characters to train adults with ASD in social cognitive domains such as emotion recognition and theory of mind (45). Significant improvements in theory of mind, emotion recognition and social skills performance were reported after the intervention (45). Similarly, the VR-based Theory of Mind Intervention (VR-ToMIS) provided an immersive social environment with virtual conversations to train theory of mind in outpatients with schizophrenia (53). The control group was able to access the same VR environment but could not interact with the avatars. Although no significant difference in quality of life was reported between the VR-ToMIS and control group, the VR-ToMIS participants demonstrated significantly greater improvements in theory of mind, pragmatic language skills, visuospatial skills and attention (49). Lastly, a study conducted in a VR CAVE-like environment trained adults with ASD to detect social cues so as to improve social perception (44). In this small-scale study, two out of the three participants reported improvements in social skills.

Two studies paired VR with Brain Computer Interface (BCI) to monitor social attention. In the Brain-Computer Interface for ASD (BCI-ASD) program, the BCI headband provided feedback on participants' attention levels while they interacted with avatars in social activities (50). These participants were adults on the autism spectrum with co-occurring diagnoses who were attending college. This study had a small sample size (*n* = 4 in each group) and no clinically meaningful change on overall adaptation to college and social adjustment was found, when compared with participants receiving a psychosocial support program (50). On the other hand, a different study which used VR-assisted BCI training for social attention found improvement in adaptive behavior composite on the Vineland Adaptive Behavior Scales but did not observe any change in joint attention among its participants with ASD after seven sessions (43).

There was only one study that used AR to train adults with ASD in public speaking. Participants performed public speaking in front of the VR system and received feedback *via* the agent's eye movement and nodding behavior (46). When compared to participants who practiced in an empty room, participants in the VR training exhibited lower anxiety and more improvement in self-confidence (46).

Lastly, one study combined VR with Cognitive Behavior Therapy (VR-CBT) to work on social avoidance in persons with schizophrenia (55). Schizophrenia participants with persistent paranoid ideation were given guided exposure to virtual social environments such as a street, bus, café, and supermarket. At follow-up, the VR-CBT participants showed improvements in social functioning and self-stigmatization, but treatment-as-usual participants did not. However, quality of life between the two groups did not differ significantly (55).

#### Quality of life

Three studies used quality of life as the sole functional outcome measurement. A small study on the use of immersive VR exposure to reduce anxiety in adults with ASD found no significant change in World Health Organization Quality of Life abbreviated (WHOQOL-BREF) scores, but five out of the eight participants reported improvements in day-to-day situations (52).

Two studies on Virtual Reality-Assisted Therapy (VRT) involved getting participants with refractory schizophrenia or schizoaffective disorders to create an avatar that best resembled the source of their residual hallucinations (48, 51). They were then facilitated by a psychiatrist to engage in a dialogue with these avatars. Statistically significant improvement in Quality-of-Life Enjoyment and Satisfaction Questionnaire-Short Form (QLESQ-SF) score was found between baseline and post-intervention (48, 51).

### Quality of studies and risk of bias assessments

#### Study design and sample size

Among the 38 selected articles, 13 were randomized controlled studies, six were quasi-experimental studies and the rest were single-arm studies. The sample size of the randomized controlled studies ranged from 4 to 64 participants, while the sample size of the quasi-experimental studies ranged from 9 to 356 participants. Among the 19 single-arm studies, nine studies had a sample size of fewer than 10 participants. Three of the single-arm studies used repeated measures while the rest adopted the pretest-posttest design.

In addition to the small sample size observed in the randomized controlled studies, it was noted that only four studies had an active comparison intervention that matched the time spent in the experimental condition. The remaining nine studies adopted either treatment-as-usual or waitlist control conditions. As for the quasi-experimental studies, almost all had some form of comparison interventions, but they were not matched to the intensity of the experimental conditions.

#### Outcome measurements used

A range of outcome measurements was used to evaluate the effectiveness of the interventions on functional outcomes. Six studies used data stored within the VR or AR games as one of the outcome measurements. Eight studies relied solely on self-developed evaluation tools that were not validated. These included questionnaire to job trainers on participants' vocational performance, checklists on number of correct steps of functional tasks, rating grid on appropriate social skills, questionnaire on participants' self-confidence in functional tasks, asking participants to recount important information or sequence of functional tasks etc. One study used the Experience Sampling Method (ESM) to track time spent with others as an objective social participation measure (47). The rest of the studies used validated outcome measurements such as the Social and Occupational Functioning Assessment Scale, Vineland Adaptive Behavior Scales, Social Functioning Scale, Personal and Social Performance scale, etc.

In addition, four studies on job interview training measured real-world functional outcomes such as successful job placements. One of the two driving simulation studies also used the attainment of a driver's permit as an outcome measure. Lastly, two studies included caregivers' reports of the participants' level of independence in community living.

#### Risk of bias assessments

ROB2 was used to evaluate the 13 randomized controlled studies and it was found that two studies had low risk of bias, seven studies had some concerns and four studies had high risk of bias. There was no particular domain which presented with several high risks.

The remaining 25 non-randomized controlled studies were assessed using ROBINS-I. Two studies were evaluated to have moderate risk of bias, 18 studies had serious risk of bias and five studies had critical risk of bias. Among the studies with serious and critical risk of bias, 19 were in domain of “bias due to confounding,” 11 were in the domain of “bias in measurement of outcomes,” and six were in the domain of “bias in selection of the reported result.”

The details of the ROB-2 and ROBINS-I assessments were presented using an adapted robvis visualization tool and found under Supplementary material.

## Discussion

This systematic review presented an overview of how VR and AR were used in psychosocial rehabilitation for adults with neurodevelopmental conditions over the past 10 years. Almost half of these studies utilized VR software programs that were presented on computer or projection screens and participants interacted with virtual characters *via* keyboard or mouse/joystick. The rest of the VR studies used head-mounted display (HMD), Cardboard Viewer, 3D glasses, CAVE system, Brain-Computer Interface (BCI), or Kinect sensors. Three studies deployed AR in functional training.

In terms of the benefits of VR or AR-assistive psychosocial rehabilitation programs, a majority of the studies reported that participants were able to execute functional tasks more accurately or with more confidence after the interventions. These functional tasks ranged from instrumental activities of daily living, job interview and customer service, to social interactions and public speaking. However, the impact on real-world functional outcomes such as community integration, job tenure and social inclusion was unclear.

### Clinical practice implications

Technological advancements in VR and AR present opportunities for clinicians to harness the unique features of VR and AR to augment traditional psychosocial rehabilitation programs. Many functional training programs involve practitioners assessing service users' functional performance through observation, followed by using a variety of approaches and methods to assist service users to perform functional activities to fulfill their valued life roles (56). Evaluating task performance in an objective and reliable manner requires close observation and calibration is a requisite for some observer-rated functional assessments (57–59). There is potential for VR to integrate sensor technology to detect body movements and gestures, videos to capture facial expressions and eye tracking to assess eye gaze, which will provide rich data on service users' functional performance in a detailed and objective manner. This will expedite functional performance evaluation and assist practitioners in setting suitable goals and intervention plans.

Some of the studies in this systematic review also made use of data from speech recognition, eye tracking motion sensors, artificial intelligence algorithms or motion-capture device in providing real-time feedback to participants during their rehabilitation (21, 34, 43). Such interactive elements enabled participants to adjust their behavior, communication and information processing strategies such that they could complete the gamified activities in a more adaptive manner. The study by Cox et al. showed that automated feedback provided by the virtual driving simulator did not differ from trainers' feedback (21). The studies on Virtual Interview Training for Transition Age Youth (VIT-TAY) also showed that a virtual job coach was able to provide additional support and feedback during the job interview training (37, 38). Therefore, it would be useful to establish the sensitivity and precision of VR or AR-enabled feedback systems and explore how these could maximize learning of new functional skills. Integrating artificial intelligence systems within VR or AR platforms may also enhance rehabilitation outcomes by matching learning activities to individual characteristics. It is unlikely for such feedback mechanisms to replace human practitioners. However, such features allow practitioners to train service users in a group and may consequently improve treatment efficiency.

Although HMDs are commonly used for full immersion in a virtual environment, only six studies in this systematic review utilized this mode of implementation. This could be due to concerns about weight and tightness of the headset or motion sickness (8). Therefore, some of the studies in this review utilized low resolution VR cardboard goggles or 3D glasses (33, 54), while others used CAVE-like systems to deliver the immersive effect (44, 52). The distinctive feature of immersive VR is its ability to provide a sense of presence with other people, by simulating real life situations such as standing in a crowded place or working in a noisy environment. Such effects could not be achieved when the virtual environment is presented on a computer screen. It will also not be feasible to set up a busy vocational training site or a public transportation network within a rehabilitation facility. Therefore, it will be useful for practitioners and researchers to harness the unique elements of VR and AR by utilizing their immersive features. The studies that utilized HMD, CAVE and 3D glasses reported generally good acceptance by participants, although there were concerns about sensory sensitivity in adults with ASD (28, 33, 53). There is a need for more research on the risk-benefit ratio of using fully immersive environment and strategies for managing motion sickness for adults with neurodevelopmental disorders.

### Addressing functional outcomes

While some studies in this systemic review used AR or VR gamified platforms to teach steps of a functional task using repeated practice (e.g., ironing clothes, taking public transportation, and wiring an electrical socket), others used the technology to coach participants to respond to different real-life scenarios using the strategy approach (e.g., solving a problem at work in a boutique, interacting with job interviewers or strangers). Both the rehearsal/repeated practice approach and strategy approach have their merits and cater to service users with different functional goals. However, the theoretical and clinical framework underpinning many of these studies were not defined, with a lack of clarity on the rehabilitation approach. It will be useful for future VR/AR-assisted psychosocial rehabilitation programs to be grounded in a conceptual framework, to guide the intervention approach.

Although a number of studies in this review implemented VR or AR interventions in a laboratory setting, there were a few studies which integrated the VR or AR interventions within an existing service. Delivering the intervention in the context of an existing program in a natural setting appeared to yield benefits in functional outcomes and could facilitate generalizability. For example, the VIT-TAY intervention was implemented for youths with ASD attending pre-employment transition services (Pre-ETS) in schools (37, 38). Teachers in the schools volunteered to be trained to facilitate the VIT-TAY sessions, which paved the way for implementing this intervention beyond the research period. Participants receiving VIT-TAY not only improved in job interview skills, but also achieved higher rates of competitive employment (37, 38). Therefore, AR/VR interventions with features that bridged ongoing psychiatric rehabilitation programs appeared to maximize outcomes. Similarly, the study by Rus-Calafell et al. involved the use of a VR Soskitrain program as an adjunct to a manualized social skills intervention for persons with schizophrenia (54). Although the study was small-scale and of a single arm pretest-posttest design, participants showed significant improvements in social functioning as measured by the Social Functioning Scale, with gains maintained at 4-month follow-up (54). When service users could apply the skills learned during AR sessions directly to their regular rehabilitation program with clear strategies to transfer learning to the real-world, the impact appeared to be greater.

Combining VR with an evidence-based intervention such as Cognitive Behavior Therapy (CBT) could also yield benefits. Pot-Kolder et al. used four virtual social environments alongside CBT to work on social avoidance in persons with schizophrenia (47). Using Experience Sampling Method (ESM) to track actual time spent with others, the authors found a treatment effect on objective real-world social participation (47). Similarly, the Virtual Reality Job Interview Training (VR-JIT) was also recently incorporated into an Individual Placement and Support (IPS) service (60). IPS is an evidence-based model of supported employment but not all service users achieve positive outcomes (61). Preliminary findings suggested that IPS non-responders benefited from VR-JIT as shown by higher employment rates within a shorter time, compared to non-responders receiving IPS only (60). Therefore, augmenting an evidence-based program with AR or VR sessions appeared to enhance the effectiveness of the program. Further research into this area is warranted.

As described earlier, many studies in this review used in-game performance or self-developed assessments to measure functional outcomes, which may not approximate real-world functioning. Some studies used performance-based assessments such as job interview role-plays with standardized actors and administered by blinded raters. Although such assessments are more structured, their validity and reliability need to be established further. With the recent emphasis on implementation science, idiographic functional outcomes such as the Goal Attainment Scale have gained traction as they may be more effective than standardized measures in capturing real-world changes in individuals (62–64) The Experience Sampling Method (ESM) is another measurement method with high ecological validity, as it utilizes technology such as mobile phones to collect participants' day-to-day report of their experiences (65). This minimizes retrospective recall bias as participants are prompted at different times of the day to report their thoughts, emotions and behaviors on the spot (65). However, considerations need to be made for participants with IDD and schizophrenia, to ensure that they can understand and feel comfortable with this form of self-reporting.

In the field of psychosocial rehabilitation, the recovery movement has brought about the conceptualization of recovery as three domains: clinical, functional, and personal (66). While clinical recovery denotes the general stabilization of mental state, functional recovery constitutes the restoration of occupational, community, and social functioning (67). Personal recovery, however, is the process of experiencing hope, empowerment, identity, connectedness, and meaning (68, 69). As the ultimate goal of psychosocial rehabilitation is to restore function and empower service users to experience personal recovery, it will be pertinent to include measurements of personal recovery when assessing long-term outcomes of VR or AR-assisted psychosocial rehabilitation programs. In addition, it was noted that only two studies in this review included caregivers' evaluation of the participants' functional independence. As many adults with neurodevelopmental conditions are supported by family or formal caregivers, it will be useful to obtain caregiver reports to corroborate self-report findings.

### Quality of selected studies

Fewer than half of the studies in this systematic review were randomized controlled studies and majority of these randomized studies used treatment-as-usual or waitlist control conditions. Although there are mixed findings on the value of an active control intervention, having a treatment-as-usual control makes it difficult to rule out placebo effects or determine if differential outcomes are due to the therapeutic elements of the experimental conditions (70–72). Assessors were also not blinded in three of these studies, which could lead to bias in measurement of outcomes.

Among the nine single-arm studies with fewer than 10 participants, some used parametric statistical analyses even though there was no indication that the data was normally distributed. Many of these studies also failed to address confounding variables and presented with bias in measurement of outcomes. To ascertain the effectiveness of AR or VR-assisted psychosocial rehabilitation programs, larger randomized controlled studies with adequate blinding and active comparison groups will be necessary.

### Study limitations and recommendations for future research

This systematic review adopted a narrative approach, as majority of the studies had small sample sizes and adopted a single arm pretest-posttest design. More robust and bigger studies are required in this area, so that meta-analyses could be conducted. Studies that delve deeper into the therapeutic components of VR or AR-enhanced psychiatric rehabilitation programs are also necessary. More research can be done on how VR or AR can facilitate strategy learning and enhance the effectiveness of evidence-based programs such as IPS. Robust studies could also be conducted on the use of VR or AR on other therapeutic interventions such as metacognition training (73, 74). In addition, the use of a consistent set of standardized functional outcome measures will enable the pooling of outcome data. With rapid advancements in the use of sensors and artificial intelligence in healthcare, it will also be useful to review their usage in psychosocial rehabilitation in the future.

This systematic review has presented preliminary promising findings on the use of virtual reality and augmented reality to enhance traditional training methods in psychosocial rehabilitation. There is a need for future research to move beyond feasibility trials and address scalability, implementation issues and dissemination across sociocultural contexts (75). As the cost of devices goes down with increased usage, it may also be useful to explore cost effectiveness of VR and AR-assisted interventions in psychosocial rehabilitation.

## Conclusion

Virtual reality and augmented reality are deployed in various ways to augment the implementation of psychosocial rehabilitation for adults with neurodevelopmental disorders. Most interventions target skills training or strategy learning in the areas of community living, work and social participation. Preliminary positive findings of their effects on functional performance were presented in this systematic review. Delivering the AR or VR intervention with features that bridged ongoing psychiatric rehabilitation programs appeared to have a positive impact on functional outcomes. In addition, combining VR or AR with an evidence-based intervention could enhance its benefits. There is potential to harness the useful features of virtual and augmented reality and integrate other technology to enhance treatment outcomes. More robust studies using ecologically valid outcome measures will be needed to establish their effects on real-world functional outcomes.

## Resource identification initiative

PROSPERO (RRID:SCR_019061).

## Data availability statement

The original contributions presented in the study are included in the article/Supplementary material, further inquiries can be directed to the corresponding author.

## Author contributions

B-LT and AM: conception and design. B-LT, JS, SY, HL, DN, and CC: article search and critical appraisal. All authors: manuscript writing and approval.
