# Supplementary material for: The use of virtual reality and augmented reality in psychosocial rehabilitation for adults with neurodevelopmental disorders: A systematic review
Source: Front Psychiatry. 2022 Dec 14;13:1055204. doi: 10.3389/fpsyt.2022.1055204 (PMC9794993; doi:10.3389/fpsyt.2022.1055204)
Supplement: Supplementary file 1 [file Data_Sheet_1.pdf]

Supplementary Material: Risk of Bias Assessments

ROBINS-I (Non-Randomized Controlled Studies) Risk of Bias Domains

	B1	B2	B3	B4	B5	B6	B7
Adery et.al. (2018)-serious risk	🔴	🟢	🟢	🟢	🟡	🟢	🟢
Amado et al. (2016) - critical risk	🔵	🔵	🟢	🟡	🟡	🔴	🟢
Amaral et al (2018) - serious risk	🔴	🟢	🟢	🟢	🟢	🟡	🟢
Baker-Ericze (2021) - serious risk	🔴	🟢	🟢	🟢	🟢	🔴	🟢
Bozgeyikli et al (2017) - critical risk	🔵	○	🟢	🟡	🟢	🟡	🔴
Bridges et al (2020) - serious risk	○	🟢	🟢	🟢	🟢	🔴	🟡
Burke et al (2018) - serious risk	🔴	○	🟡	🟢	🟢	○	🟡
Burke et al (2021) - moderate risk	🟡	○	🟢	🟡	🟢	🟢	🟡
Chang et.al. (2013)- critical risk	🔵	🟡	🟢	○	🟢	🔴	🔴
Cox (2017) - serious risk	🔴	🟢	🟢	🟢	🟡	🟡	🟡
Dellazizzo et al (2020) - serious risk	🔴	🟡	🟢	○	🟢	○	🟢
de la Torre-Luque et.al. (2017)-serious risk	🔴	🟢	🟢	○	○	🔴	🟡
Giachero et al (2021)-serious risk	🔴	🟢	🟢	🟢	🟡	🔴	🟢
Jacques et al (2018)-critical risk	🔵	○	🟢	🟢	🟢	🔴	🟢
Jeon et al (2021)- serious risk	🟢	🟢	🟢	🟢	○	🔴	🟢
Kandalaft et al (2013)- serious risk	🔴	🟢	🟢	🟢	🟢	🟡	🟢

	B1	B2	B3	B4	B5	B6	B7
Kuper et al. 2020-serious risk	Serious risk	Serious risk	Moderate risk	Serious risk	Low risk	No information	No information
Maskey et al (2019)- serious risk	No information	Low risk	Moderate risk	Low risk	Low risk	Moderate risk	Serious risk
Miller et al (2020)- critical risk	Serious risk	Critical risk	Moderate risk	No information	Serious risk	Serious risk	Serious risk
Panerai et al (2018)- serious risk	No information	No information	Serious risk	Moderate risk	Low risk	Moderate risk	Moderate risk
Rus-Calafell et al. (2013)- serious risk	Serious risk	Moderate risk	Low risk	Moderate risk	No information	Serious risk	Serious risk
Saiano et.al. (2015)-serious risk	Serious risk	Low risk	Low risk	Low risk	Low risk	Serious risk	Serious risk
Simões et al (2018)- serious risk	Serious risk	Moderate risk	Low risk	Serious risk	Moderate risk	Moderate risk	Low risk
Smith et al. (2022)- moderate risk	Moderate risk	Low risk	Low risk	Low risk	Moderate risk	Low risk	Low risk
Sohn et al. (2016)-serious risk	Serious risk	Low risk	Low risk	Low risk	Low risk	Moderate risk	Low risk

Domains:
B1: Bias due to confounding
B2: Bias in selection of participants
B3: Bias in classification of interventions
B4: Bias due to deviations from intended interventions
B5: Bias due to missing data
B6: Bias in measurement of outcomes
B7: Bias in selection of the reported result

Level of Risk:

Low risk

Moderate risk

Serious risk

Critical risk

No information on which to base judgment about risk

ROB-2 (Randomized Controlled Studies) Risk of Bias Domains

	B1	B2	B3	B4	B5
Camera et.al. (2021)-some concerns	🟢	🟡	🟢	🟡	🟢
Du Sert et al (2018)-high risk	🟢	🔴	🟡	🔴	🟡
Humm et al. (2014)-high risk	🟡	🔴	🔴	🟢	🔴
Kumazaki et al. 2020-high risk	🟢	🔴	🟢	🔴	🔴
Pot-Kolder et.al. (2018)-some concerns	🟢	🟢	🟢	🟡	🟢
Smith et.al. (2014)-some concerns	🟡	🟡	🟢	🟢	🟢
Smith et al. 2015)-some concerns. *Brief Report: Vocational Outcomes for Young Adults with Autism Spectrum Disorders at Six Months After Virtual Reality Job Interview Training*	🟡	🟡	🟢	🟢	🟢
Smith et al. (2015)-high risk *Virtual Reality Job Interview Training and 6-Month Employment Outcomes for Individuals with Schizophrenia Seeking Employment.*	🟡	🟡	🔴	🟢	🟢
Smith et al. (2021)-some concerns	🟡	🟢	🟢	🟢	🟢
Strickland et.al. (2013)-some concerns	🟢	🟢	🟢	🟡	🟢
Tsang & Man (2013)-low risk	🟢	🟢	🟢	🟢	🟢
Vass et al. (2020)-low risk	🟢	🟢	🟢	🟢	🟢
White et al. (2016)-some concerns	🟡	🟢	🟢	🟢	🟡

Domains:
B1: Bias due to randomization
B2: Bias due to deviations from intended intervention
B3: Bias due to missing outcome data
B4: Bias due to measurement of outcome
B5: Bias due to selection of reported results

Level of Risk:

🟢 Low risk

🟡 Some concerns

🔴 High risk
